# Potential Application Performance of Hydrochar from Kitchen Waste: Effects of Salt, Oil, Moisture, and pH

**DOI:** 10.3390/toxics11080679

**Published:** 2023-08-08

**Authors:** Xuesong Su, Jizu He, Muhammad Amjad Khan, Kenlin Chang, Yin Liu, Genmao Guo, Xiaohui Li, Fangming Jin, Meijuan Kuang, Shaban Gouda, Qing Huang

**Affiliations:** 1 School of Ecology & Environment, Hainan University, Haikou 570228, China; 2 Institute of Environmental Engineering, Department of Public Health, National Sun Yat-Sen University, Kaohsiung 804, Taiwan; klchang@mail.nsysu.edu.tw; 3 Hainan Inspection and Detection Center for Modern Agriculture, Haikou 570100, China; 4 Hainan Pujin Environmental Engineeering, Haikou 570100, China; 5 Agricultural and Biosystems Engineering Department, Faculty of Agriculture, Benha University, Toukh 13736, Egypt

**Keywords:** hydrochar, adsorption, combustion, resource utilization, food waste

## Abstract

The surge in kitchen waste production is causing food-borne disease epidemics and is a public health threat worldwide. Additionally, the effectiveness of conventional treatment approaches may be hampered by KW’s high moisture, salt, and oil content. Hydrothermal carbonization (HTC) is a promising new technology to convert waste biomass into environmentally beneficial derivatives. This study used simulated KW to determine the efficacy of hydrothermal derivatives (hydrochar) with different salt and oil content, pH value, and solid-liquid ratio for the removal of cadmium (Cd) from water and identify their high heating value (HHV). The findings revealed that the kitchen waste hydrochar (KWHC) yield decreased with increasing oil content. When the water content in the hydrothermal system increased by 90%, the yield of KWHC decreased by 65.85%. The adsorption capacity of KWHC remained stable at different salinities. The KWHC produced in the acidic environment increases the removal efficiency of KWHC for Cd. The raw material was effectively transformed into a maximum HHV (30.01 MJ/kg). HTC is an effective and secure method for the resource utilization of KW based on the adsorption capacity and combustion characteristic indices of KWHC.

## 1. Introduction

Human population growth and improved living standards have resulted in a sharp rise in kitchen waste (KW) production. Foodborne illnesses caused by KW are still very prevalent and public, which has a huge impact on global public health (for example, African swine fever and recycling of gutter oil). Although strict regulations and advanced food safety systems regulate the disposal of kitchen waste, millions of people still get sick, and thousands of people die of related foodborne illnesses every year [1]. In addition, the public panic caused by COVID-19 may have resulted in an increase in online food shopping and disruption of the food supply chain, both of which further expanded the production of KW and put additional pressure on waste management systems [2]. Many countries are looking for new waste disposal methods and systems to cope with the environmental pressures and potential risks posed by KW during the pandemic [3]. KW is typically characterized by a high oil, salt, and moisture content due to human dietary habits and practices [4]. These characteristics severely reduce the effectiveness of conventional waste treatment techniques, including landfilling, incineration, and biological treatments, which can also cause secondary sources of environmental pollution [5]. It has been reported that the salt in KW is one of the reasons for the presence of dioxins in waste incineration gas; similarly, the oil in KW forms leachates [6]. The chemical complexity and biological toxicity of landfill leachate eventually migrate into soil and water, endangering public health and environmental security [7]. Furthermore, the high moisture content in KW reduces the efficiency of biological approaches (e.g., anaerobic digestion, composting) and increases operating costs [8]. Appropriate disposal of KW is the key issue to maintaining environmental sustainability and is crucial to its safe and effective disposal and utilization. It has been a challenge for researchers to find a more rational resource treatment method to assign the value of KW during the epidemic.

Hydrothermal carbonization (HTC), a new method of waste resource utilization, has gained increasing attention from researchers in recent years due to the lack of restrictions on the water content of the raw material and the fact that it does not require pre-drying, making it more suitable for biomass waste. At a certain temperature and self-generated pressure, HTC can quickly convert organic matter into carbon-rich products using water as a solvent and reaction medium [9]. Prior research has discovered that COVID-19 was inactivated by heating at 56 °C for 30 min [10]. In addition, early articles demonstrated that the HTC reaction temperature is relatively high (150–350 °C) and can be used for sterilization to reduce pathogens and prevent infection [11]. Therefore, HTC is a more suitable KW disposal method to reduce potential risks for public health and safety. Meanwhile, as heavy metal adsorbents and fuel replacements, hydrothermal derivatives also offer extraordinarily high value for environmental applications [9].

Cadmium (Cd) is one of the most toxic heavy metals that can contaminate the soil and build up in plants, which poses a risk to humans [12,13]. The scarcity of fossil fuels and the inability of energy utilization structural monophyly to maintain globally growing fuel demand and environmental sustainability, as well as the search for energy alternatives, are imminent [14]. While KW hydrothermal derivative (hydrochar) can not only be used to recover Cd-contaminated wastewater due to its porous and carbon-rich properties, it can also be made as an alternative to traditional fuels, alleviating pollution of the environment and global energy problems [15].

HTC offers an alternative for rapid, safe, and resource-based treatment of KW. However, when applying HTC to KW treatment, the material specificity of its high salt and high oil content, which affects the mechanism of the physicochemical properties and application performance of HTC process and its derivatives, is still not clear; pH and solid-liquid ratio were taken as significant parameters to influence the hydrothermal reaction, and the mechanism of their effects on the application of KW to HTC still deserves further exploration [16].

In this study, HTC is employed to further improve the environment while easing the burden of solid biomass waste disposal. The objectives of the present study are: (1) to produce kitchen waste hydrochar (KWHC) from KW with various contents of salt and oil, pH, and solid-liquid ratios; (2) to identify the effects of KWHC prepared with different oil, salt, pH, and solid-liquid ratios on the Cd adsorption capacity and the combustion characteristics of KWHC; and (3) to explore the correlation between oil, salt, and the application performance of hydrothermal derivatives of KW.

## 2. Materials and Methods

### 2.1. Materials

In this study, rice, eggs, and lettuce were used as typical components representing carbohydrates, protein, and cellulose as a simulated KW, and the proportion of components used was mentioned in [17]. The solid-liquid ratio of 1:5 provided the highest yield of hydrochar during the pre-experiment; hence, it was chosen as the blank control (CK). On the other hand, four treatments were prepared using different salt concentrations (S), solid-liquid ratios (R), pH (P), and oil concentrations (O). Moreover, various contents of edible salt (0.5%, 1%, 5%, and 10%) were added to the simulated KW afterwards, referred to as S1, S2, S3, and S4, respectively. Similarly, the simulated KW and water with different solid-liquid ratios of 1:1, 1:5, 1:10, and 1:20 were prepared afterwards and named R1, R2, R3, and R4, respectively. For pH, solutions of 3, 5, 8, and 10 pH values were adjusted with NH_3_•H_2_O and HNO_3_ to make hydrochar, and different oil contents of 10%, 20%, 30%, and 40% were adjusted using cooking oil labeled P1, P2, P3, and P4, and O1, O2, O3, and O4, respectively. All treatments are listed in Table 1.

### 2.2. Hydrothermalcarbonization of Synthetic KW

All the prepared treatments were placed in a high-pressure hydrothermal reactor (KH, Reactor, Wuhan, China) at 200 °C for 10 h. After cooling to room temperature, the solid-liquid parts were separated by 0.45 µm filter paper. Finally, hydrochar was dried at 80 °C overnight to a constant weight.

### 2.3. Adsorption Capacity of KWHC

#### 2.3.1. Sorption Kinetics

Kinetic models provide an explanation of adsorbent behavior. To evaluate the adsorption capacity, 0.2 g of each KWHC sample was added to a centrifuge tube containing 40 mL of a 50 mg/L Cd solution. For 10, 20, 30, 90, 120, 150, 180, 240, and 300 min, the centrifuge tubes oscillated at a temperature of 30 °C at 180 rpm/min [18]. Pseudo-first-order (PFO) and pseudo-second-order (PSO) models were used to describe the adsorption behavior.

The PFO and PSO kinetic models are represented in Equations (1) and (2), respectively, as follows:

(1) $$qt=qe(1-\exp(-k_{1}t)),$$

(2) $$qt=\frac{k_{2}{qe}^{2}t}{1+k_{2}qet},$$

where qe and qt are the adsorption capacities at equilibrium (mg/g) and time t (h), respectively, and k_1_ and k_2_ are the kinetic adsorption rate constants for the PFO and PSO models (h^−1^), respectively.

#### 2.3.2. Sorption Isotherms

In the KWHC adsorption experiments, the Cd (II) solution concentrations were set at 10, 20, 50, 100, 200, and 500 mg/L. KWHC (0.2 g) and 40 mL of Cd (II) solutions were added into 50 mL centrifuge tubes [19]. The samples were shaken at 160 r/min at 25 ℃ for 24 h to achieve adsorption equilibrium [20]. The Langmuir and Freundlich models were used to describe adsorption behavior.

The Langmuir and Freundlich models are represented in Equations (3) and (4), respectively, as follows:

(3) $$\frac{Ce}{qe}\;=\frac{1}{qmKl}\;+\frac{Ce}{qm},$$

(4) $$lgqe=lgKF+\frac{1}{n}lgCe,$$

where qm is the theoretical maximum adsorption capacity (mg/L), Ce is the equilibrium concentration (mg/L), Kl is the Langmuir adsorption coefficient (L/mg), KF is the Freundlich adsorption coefficient (L/g), and n is the adsorption strength (-).

The Cd (II) concentration was measured after the supernatant was filtered through a 0.45 µm filter membrane. An atomic absorption spectrometer (TAS-990 SUPER AFG, Beijing, China) was used to analyze Cd (II) concentrations.

The adsorption quantity of KWHC was calculated according to Equation (5):

(5) $$q=\frac{(C_{0}-C_{1})\times V}{m},$$

where q is the adsorption quantity of KWHC (insert unit), C_0_ and C_1_ are the initial and equilibrium concentrations of Cd (II) (mg/L), respectively, m is the adsorbent mass (g), and V is the adsorption solution volume (L). 

### 2.4. Thermogravimetric Analysis of KWHC

The combustion characteristics were tested by a thermal gravimetric analyzer (TG, 209 F3, China). An alumina sample cup containing 10 mg of sample was loaded into the analyzer. The sample was heated to 40–105 °C for 5 °C per min, held at isothermal conditions for 10 min to remove residual water, and then heated to 800 °C at a rate of 20 °C per min in an N_2_ atmosphere to obtain decomposition data [21].

To provide further information on the combustion activity of the samples, Ti and Tb were used to calculate the combustibility index (S) through Equation (6) to reflect the overall combustion rate:

(6) $$S=\frac{{\left(dw/dt\right)}_{max}\times {\left(dw/dt\right)}_{mean}}{{T_{i}}^{2}{\times T}_{b}},$$

where (dw/dt)_max_ and (dw/dt)_mean_ correspond to the maximum and average rate of weight loss (% wt./min), respectively, and T_i_ and T_b_ are the ignition temperature and burnout temperature (°C), respectively.

In addition to peak deconvolution, T_i_, peak temperature (Tp), and Tb were determined from difference thermogravimetry (DTG) profiles using the TG-DTG tangent method [22].

### 2.5. Chemical Analysis of KWHC

Using an elemental analyzer (Elementar Vario EL cube, Germany), the contents of carbon (C), oxygen (O), hydrogen (H), and nitrogen (N) in hydrochar were measured. The higher heating value (HHV) of the samples was calculated according to Equation (7) [23].

(7) HHV=0.3383×C+1.422×(H−O/8),

where HHV is the higher heating value (MJ/kg), C, H, and O are the mass contents of carbon, hydrogen, and oxygen (wt.%), respectively.

### 2.6. Analytical Methods

The functional groups were analyzed by Fourier Transform Infrared Spectrometry (FTIR) (FTIR-650, Andong, China). The potassium bromide (KBr) and hydrochar samples were mixed at a ratio of 1:100 and pressed into tablets. The spectral range measured was 4000–400 cm^−1^. The mineral compositions were measured by X-ray diffraction (XRD, DX-2700BH, China).

### 2.7. Statistical Analysis

Statistical analyses were performed by the analysis software SPSS v.18.0 for Windows (IBM-SPSS, Statistics 22, Armonk, NY, USA). Means were compared by single-factor analysis of variance (significant difference, *p* < 0.05). The structural equation model showed the direct and indirect effects of salt, oil, pH, and solid-liquid ratio on the yield, adsorption, and calorific value of KWHC. “*”, “**”, and “***” indicate significant differences at *p* < 0.05, *p* < 0.01, and *p* < 0.001, respectively. The black solid arrows and the gray dashed arrows represent a positive and negative relationship, respectively.

## 3. Results

### 3.1. Effects of Salt, Oil, pH, and Solid-Liquid Ratio on KWHC Productivity

**Figure 1:**
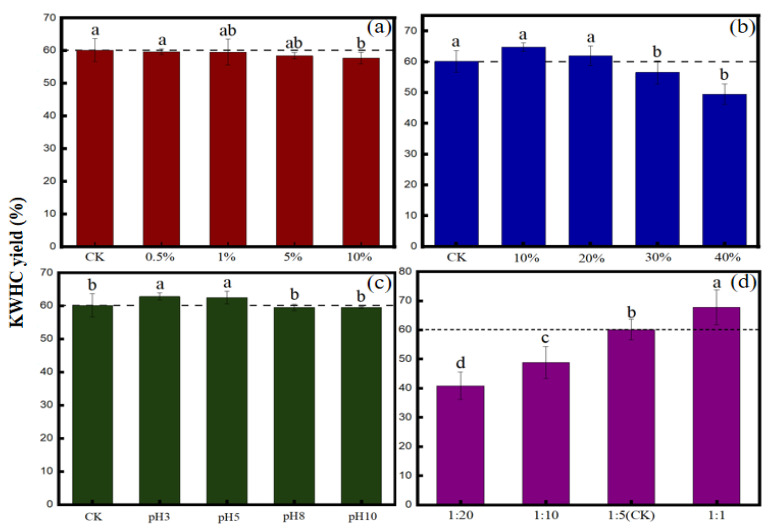
The productivity of KWHC as affected by different treatments: (**a**) salt contents, (**b**) oil contents, (**c**) pH, and (**d**) solid-liquid ratios. For an experimental variable, different letters indicate significant differences in concentration changes between the same treatments (*p* < 0.05).

The productivity of KWHC was stable with increasing salt content, except in the treatment with 10% salt content, where the KWHC productivity slightly decreased by 4.11% compared to CK (Figure 1a). According to the results of the present study, the oil content had a negative relationship with the KWHC yield. The KWHC yield decreased from 64.80% to 48.52% as the oil content of KW increased from 10% to 40% (Figure 1b). The productivity of KWHC in the acid solutions (pH 3 and pH 5) was not significantly different compared with the blank control, and there was also no observable yield difference under alkaline conditions (Figure 1c). With the increase in solution pH from 3 to 8, the productivity of KWHC decreased slightly by 5.21%. With the increase in water content in the hydrothermal reaction system, the yield of hydrochar showed a declining trend (Figure 1d). When the hydrothermal system’s water content increased from 50% to 95%, the production of KWHC decreased by 65.85%.

### 3.2. Elemental Analysis of the KWHC

In KWHC, the C and N contents decreased by 6.81% and 13.26% after HTC, when the salt content increased from 0.5% to 10%. The salt had no striking effect on the H content. The oil content strongly affected the elemental composition of KWHC. As the oil content increased, the C and N contents decreased by 31.20% and 37.69%, respectively. The carbon content of KWHC decreased with decreasing pH in the range of 5 to 7. The C/N ratio in KWHC increased with increasing pH (3–10). The solid-liquid ratio of 1:5 KWHC resulted in the highest C/N ratio (Table 2).

### 3.3. Adsorption of Cd (Ⅱ) on KWHC with Different Treatments

**Figure 2:**
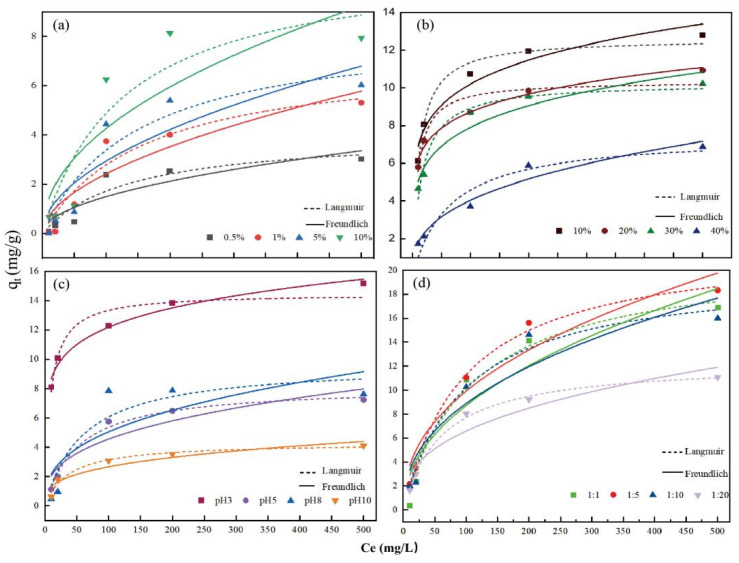
Langmuir and Freundlich adsorption models of KWHC. Notes: (**a**) salt contents; (**b**) oil contents; (**c**) pH; and (**d**) solid-liquid ratios.

Increasing salinity increased the Cd (II) removal efficiency of KWHC by 10.76%, which showed that high salt content in KW promoted the adsorption performance of KWHC (Figure 2a). With increasing oil content, the adsorption quantity and removal efficiency of hydrochar in the solution with a high Cd (II) concentration decreased by 12.16%, which means that the oil content had little adverse effect on the adsorption capacity of the hydrochar (Figure 2b). The adsorption capacity of KWHC increased with the increase in pH of the aqueous Cd (Ⅱ) solutions. The removal capacity of hydrochar for Cd (Ⅱ) increased by 12.71% with the increase in the solution pH from 3 to 10 (Figure 2c). Increased water content in the hydrothermal process reduced the Cd (II) adsorption capacity of KWHC. The treatments prepared with a solid-water ratio of 1:5 had the highest adsorption capacity out of all the treatment groups. The increase was 28.22% compared to the lowest adsorption group (1:20 solid-water ratio) (Figure 2d).

### 3.4. Combustion Characteristics Analysis

#### 3.4.1. HHV Analysis of KWHC

The HHV of CK KWHC was 26.376 MJ/kg. It has a high HHV compared to similar waste hydrochar. For example, the HHV of sludge hydrochar was 9.04 MJ/kg, and with the addition of 10% NaCl, its HHV decreased to 6.92 MJ/kg. The hydrochar HHV obtained in the co-hydrothermal experiment of rape straw and microalgae was 22.30 MJ/kg. The HHV of cattle manure hydrochar was 17.21 MJ/kg. With increased salt content, the calorific value of hydrochar decreased slightly. The HHV of KWHC was only reduced by 7.8% even when the salt content reached 10%. The HHV of KWHC decreased significantly by 35.07% when the oil content increased. However, even when the oil fraction was as high as 40%, hydrochar still exhibited a high calorific value (19.49 MJ/kg). Regarding pH, the HHV of KWHC was slightly enhanced (4.12%) under alkaline conditions compared to acidic conditions. As the amount of water in the hydrothermal process increased, the KWHC’s HHV marginally increased (3.8%) (Table 3).

The results of the present study revealed that salt had no significant impact on the HHV of KW hydrothermal derivatives. However, KW with a high oil content still exhibits excellent combustion performance after hydrothermal treatment. The phenomenon of the high heating value increasing with the increase in pH corresponds to increased C content and decreased O and H contents in the KWHC. It has been reported that the ratio of C increases with the increase in alkalinity, indicating that carbonization is intensified [24]. The contents of O and H decreased due to the formation of CO_2_ and H_2_O by dehydration and decarboxylation reactions during HTC (Table 3) [25]. This occurred because increasing the water concentration accelerates the hydrolysis reaction during the hydrothermal process. The rapid breakdown reaction brought on by increased water content is another factor contributing to the increase in the HHV of hydrochar (Table 3) .

All treatments were found to have extremely high HHV (19.49–30.01 MJ/kg) (Table 3). As Zhang et al. (2020) illustrated, the increase in HHV may be caused by the cleavage of low-energy chemical bonds and the formation of high-energy chemical bonds in hydrochar during HTC [26]. The outcome showed that even when the KW has a high level of salt and oil, the hydrothermal process can still turn biomass waste into solid fuel with good calorific value. The pH and water contents in the hydrothermal process do not need to be tightly controlled to maintain the HHV of hydrochar (Table 3). Considering that KW cannot be adequately treated with conventional procedures due to its high oil, salt, and moisture content, the harmful effects of these components can be effectively avoided using the hydrothermal approach. Compared to other treatment options, this treatment has a distinct benefit regarding renewable energy.

#### 3.4.2. Thermogravimetric Analysis of KWHC

**Figure 3:**
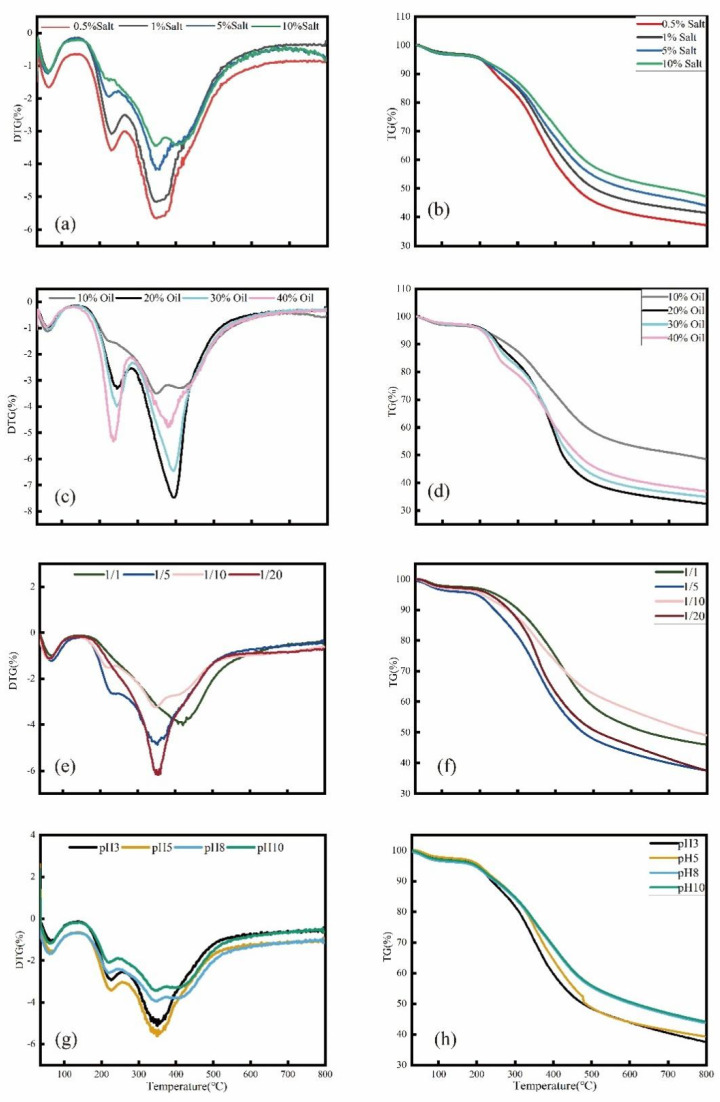
TG and DTG curves of KWHC combustion: (**a**,**b**) salt contents, (**c**,**d**) oil contents, (**e**,**f**) pH, and (**g**,**h**) solid-liquid ratios.

Using the weight loss peaks in the combustion DTG curve as a guide, the combustion of KWHC may be separated into three steps. The first stage is the drying stage, where water evaporates completely before 120 °C [27]. The second stage is the loss of volatile matter, and the combustion stage occurs at about 240 °C. There is a noticeable weight loss peak due to a large number of volatile compounds burning and the heat produced by combustion intensifying the combustion response [28]. The DTG curve exhibits a second conspicuous weight loss peak in the third stage, which corresponds to the combustion stage at roughly 380 °C. The primary cause is the transformation of the chemicals for the second-stage mass loss into those for the third-stage combustion Figure 3a,c,e,g. Additionally, fixed carbon combustion occurring during the hydrothermal reaction is the real source of the mass loss in the third stage [29].

#### 3.4.3. FTIR Analysis of KWHC

**Figure 4:**
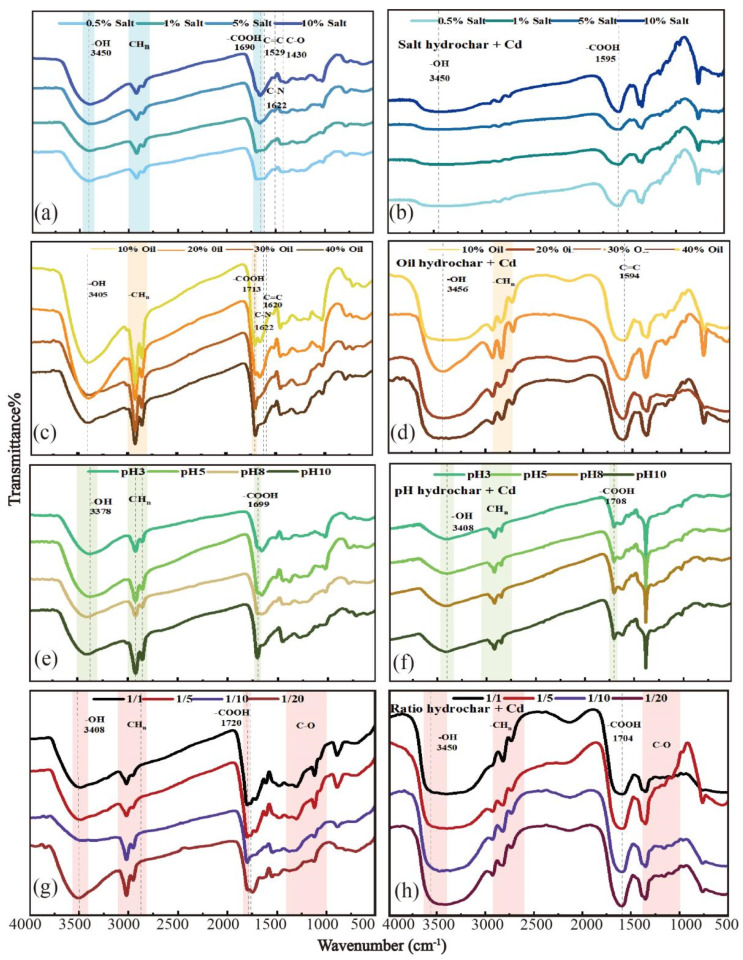
FTIR spectra for KWHC prepared under different conditions before and after Cd (Ⅱ) adsorption; (**a**,**c**,**e**,**g**) represent different treatment groups of KWHC before adsorption; and (**b**,**d**,**f**,**h**) represent different treatment groups of KWHC after adsorption.

The adsorption capacity of hydrochar is related to surface complexation and ion exchange by surface functional groups [30]. The spectra of KWHC before the adsorption tests are depicted in Figure 4a–d). The -OH stretching vibration is close to 3400 cm^−1^ [30]. The -COOH vibration absorption peak around 1705 cm^−1^ is mainly due to absorption by lipids and carboxylic acids [30]. Clearly, the peaks of hydroxyl and carboxyl groups changed significantly after the adsorption of Cd, and the peaks of -OH and -COOH were shifted from 3405 cm^−1^ to 3456 cm^−1^, and 1705 cm^−1^ to 1751 cm^−1^, respectively, as shown in Figure 4e–h. Consequently, these two functional groups play a critical role in the adsorption reaction due to their ability to interact strongly with Cd (II) through ion exchange and surface complexation [31]. The C-O vibration bands appear at 1430 cm^−1^ and in the region from 1000 to 1300 cm^−1^. Interestingly, the C-O peak shifted from 1300 cm^−1^ to 1351 cm^−1^ in Figure 4e,h), suggesting that the C-O band also has a noticeable effect on adsorption by hydrochar prepared at different solid-water ratios. The bands between 1000 and 1300 cm^−1^ for hydrochars prepared at 1:1 and 1:5 solid-liquid ratios become more intense. Vibrational changes in these functional groups may be related to the high adsorptivity of hydrochar with a low water content.

HTC’s solid products have excellent chemical and thermal stability, and thermogravimetric analysis and FTIR results confirm that there will be little secondary pollution during the recycling of KW [32].

#### 3.4.4. XRD Analysis of KWHC

**Figure 5:**
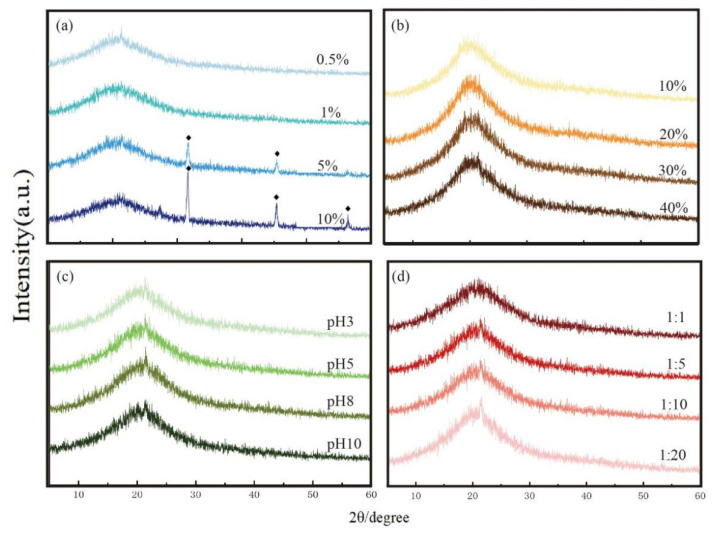
X-ray diffraction of hydrochar: (**a**) salt contents, (**b**) oil contents, (**c**) pH, and (**d**) solid-liquid ratios. ♦ represents sodium chloride (NaCl).

The graphitization degree of KWHC can be characterized by XRD and could have an impact on the combustion reaction. According to XRD data, the graphite structure of hydrochar was not significantly altered by the oil or salt concentrations of KW. At 2θ values of about 20°–30°, the typically sharp and narrow peak of graphite vanished and was replaced by a broad peak (Figure 5). This means that the development of this peak demonstrated the extent of carbonization and the presence of the graphite structure in the hydrochar [33]. Liang et al. (2022) reported that hydrochar can be utilized as a replacement for conventional fuels since it has a structure that is comparable to that of bituminous coal [15]. The XRD results indicated that variations in oil, salt, water, and pH will not significantly affect the flammability of the hydrothermal derivatives of KW. This further demonstrates the high thermal stability of the KW hydrothermal derivatives.

## 4. Discussion

### 4.1. Effects of Salt, Oil, pH, and Solid-Liquid Ratio on the Productivity of KWHC

#### 4.1.1. Effect of Salt Content on the Productivity of KWHC

Salt slightly inhibits the production of KWHC. According to Ma et al. (2021), NaCl can catalyze the release of more organic compounds into the aqueous phase during the hydrothermal process, and Cl**^−^** can disrupt the ether bonds between monosaccharides, catalyzing the hydrolysis of cellulose and causing a decrease in solid content [34]. It was assumed that the causes of this phenomenon were the same in our case. The salt concentration did not significantly affect the yield of KWHC (*p* > 0.05).

#### 4.1.2. Effect of Oil Content on Productivity of KWHC

The production of carbon decreases with an increase in oil content. The possible explanation for this reduction in yield may lie in the fact that lipids undergo complex processes to produce monoglycerides and fatty acids by hydrolysis and oxidation reactions at high temperatures [35]. Some volatile lipids evaporate or turn into gas, lowering the amount of fat in the solid phase and the solid mass [36]. Lipids also speed up the amidation reaction and create numerous liquid phases, lowering the yield of hydrochar [12].

#### 4.1.3. Effect of the pH of the Solution on the Productivity of KWHC

The production of KWHC is higher under acidic conditions. It was reported that acid can drive the self-assembly of F127 micelles and pentose in cellulose to form a stable structure and contribute to the formation of hydrochar [37]. Meanwhile, the fatty acids produced during protein hydrolysis can aid in the breakdown of holocellulose into hydrochar and produce chemicals that prevent further degradation of biomass waste [38]. Similarly, alkaline catalysts prevent the development of hydrochar, and an alkaline environment tends to reduce hydrochar generation [39].

#### 4.1.4. Effect of the Solid-Liquid Ratio on the Productivity of KWHC

High moisture content reduces the yield of KWHC. This can be accounted for by the faster conversion of wet biomass at a lower solid concentration, which leads to the almost total dissolution of the organic matter with minimal residue [13]. Additionally, when the water content drops, subcritical water results in significant carbonization, leading to the development of additional hydrochar [40]. According to reports, the solid load should be as large as feasible to enhance the production of hydrochar [41]. In comparison to salt, oil, and pH, the results demonstrated that the solid-to-water ratio has the most significant impact on the yield of hydrochar.

### 4.2. Effects of the Salt, Oil, pH, and Solid-Liquid Ratio on Elemental Analysis of KWHC

#### 4.2.1. Effects of Salt on the Elemental Analysis of KWHC

The C, N, and H contents decrease with increasing salt content. Similar results were obtained by Ma et al. (2021), who reported that the elemental contents of hydrochar with a high salt content were relatively lower than those of hydrochar without salt or with a low salt content [24]. This suggests that NaCl can catalyze hydrolysis and decarboxylation during hydrothermal carbonization, bringing more organic matter into the liquid phase and lowering the amount of solid phase produced [42].

#### 4.2.2. Effects of Oil on the Elemental Analysis of KWHC

Increasing the oil content reduces the nitrogen content. This may be because proteins participate in amination and Maillard reactions; adding lipids weakens the intensity of the Maillard reaction, which results in a weaker N-heterocyclic structure. At the same time, enhancement of the amination reaction will promote decarboxylation and deamination reactions, further reducing the N content in hydrochar [43]. The O/C ratio showed a distinct increasing trend with the increase in oil content, which implied that the decarboxylation reaction might be promoted by oil in the hydrothermal reaction [12].

#### 4.2.3. Effects of pH on the Elemental Analysis of KWHC

Under the high temperature and alkaline conditions, the C/N ratio in KWHC increased with increasing pH (3–10) due to the loss of N [44,45].

#### 4.2.4. Effects of the Solid-Liquid Ratio on the Elemental Analysis of KWHC

The solid-liquid ratio of 1:5 KWHC resulted in the highest C/N ratio. This finding agreed with the results of Kavindi et al. (2022). The content of N increased by 70% with the increase in water content (Table 2). Xu et al. (2021) explained that the increase in N content might be caused by the aggravation of the decarbonization and dehydration reactions by water [45].

### 4.3. Adsorption of Cd (Ⅱ) on KWHC with Different Treatments

#### 4.3.1. Effects of Salt on Adsorption of Cd (Ⅱ) on KWHC

Two explanations may be given for the enhancement of the adsorption capacity of KWHC by raising the salt content. Firstly, the Na^+^ ions will increase with increased salt addition. However, Na^+^ does not compete with Cd (II) for negatively charged adsorption sites, and the electrostatic repulsion between the two positive ions does not hinder the loading of Cd (II) at the surface sites. The mechanism by which Cd (II) is removed is primarily inner sphere complexation [4,46]. Secondly, in desalination techniques, for most capacitive deionization (CDI) studies, an increase in NaCl concentration will lead to an increase in salt adsorption capacity (SAC), which could affect the specific surface area of carbon materials [47]. Moreover, salinity has little effect on the hydrothermal treatment of KW, which demonstrates that the hydrothermal approach can effectively limit the adverse impact of high salt content in KW and provide steady Cd (II) adsorption performance in hydrothermal products.

#### 4.3.2. Effects of Oil on Adsorption of Cd (Ⅱ) on KWHC

According to the findings of this study, it can be assumed that the hydrothermal approach can effectively reduce the drawbacks brought on by high oil content and maintain the Cd (II) adsorption efficiency of hydrochar at a generally steady level. Jin et al. (2016) showed a successful hydrolysis of organics in KW to small molecules and inorganic species after thermal treatment, and a considerable portion of the oil fraction was dissolved from the KW into the liquid phase [26]. Hydrothermal procedures have better degreasing efficacy than conventional pre-treatments, which many researchers have employed to effectively de-oil KW [7].

#### 4.3.3. Effects of pH on Adsorption of Cd (Ⅱ) on KWHC

This phenomenon of Cd (II) adsorption increasing with the decrease in solution pH can also be explained by the competition between metal ions and protons for binding sites decreasing with increasing pH, so that metal ion binding sites become easier to obtain [47]. Cui et al. (2016) speculated that the protonation/deprotonation of surface functional groups on the hydrochar is influenced by the water’s pH, which in turn impacts the adsorption reaction [38]. The presence of H_3_O^+^ or H^+^ ions, which compete with Cd (II) for adsorption sites, may cause a reduction in adsorption capacity at low pH [48]. Another explanation could be that the acidic functional groups are protonated at lower pH levels, which results in widespread metal ion repulsion and less Cd (II) adsorption [49]. Increasing pH increases the adsorption capacity due to the cationic -π interaction between Cd (II) and hydrochar. This is attributed to the deprotonation of carboxyl and hydroxyl groups, where the weak π electron donor of the -COOH functional group is deprotonated to form -COO-, a strong π electron donor [37,39,48,49].

#### 4.3.4. Effects of Solid-Liquid Ratio on Adsorption of Cd (Ⅱ) on KWHC

The results show that the water content is important for the mechanism of HTC because the carbonization process is accelerated by water [50]. On the one hand, water acts as a favorable medium for the ionic interaction of nonpolar organic molecules during the HTC process. Particularly during the hydrolysis process, high temperatures cause the break of hydrogen bonds, increasing the amount of H^+^ and OH^−^ ions [41,51]. Due to competition for adsorption sites and a decrease in adsorption capacity, a high water content will result in a high H^+^ ion content [51]. On the other hand, water molecules may participate in carbonization reaction steps as reactants or products, facilitating hydrolysis, hydrogen exchange, condensation, and cleavage. However, the amount of water that is needed for these functions is minimal. With lower solid-water ratios, there have been reports that the hydrochar surface can form more oxygen-containing functional groups because the hydrochar surface is susceptible to hydrolysis. This may result in an increase in the adsorption capacity of the hydrochar [52]. It can be inferred from the experimental results that the use of an appropriate solid-liquid ratio can effectively increase the adsorption efficiency of hydrochar. However, the influence of water content on the Cd (II) adsorption performance of hydrochar needs further study.

According to the analysis of the adsorption efficiency of KWHC, the removal efficiency of KWHC is higher when the Cd (II) content in solution is low, while it is lower when the Cd (II) concentration is high. The KWHC is more suitable for adsorbing and removing low Cd (II) concentrations from aqueous solutions.

### 4.4. Adsorption Mechanism of KWHC

#### 4.4.1. Sorption Isotherms

According to the analysis of the fitting degree of the equilibrium concentration of Cd (II) adsorption and the adsorption capacity of KWHC using the Langmuir adsorption model and the Freundlich models, the fitting coefficient of Langmuir is higher than that of Freundlich, as illustrated in Figure 2 and Table 4. Therefore, the isothermal adsorption model of KWHC is more suitable for the Langmuir model, which proves that the adsorption mechanism of KWHC for Cd (II) is a single-layer homogeneous adsorption process [53,54]. This further confirms that KWHC has a greater affinity for Cd (II), and stable inner spherical complexes can be generated inside KWHC to enhance the coordination of Cd (II) with surface functional groups (-OH, -COOH) [47,54].

#### 4.4.2. Sorption Kinetics

**Figure 6:**
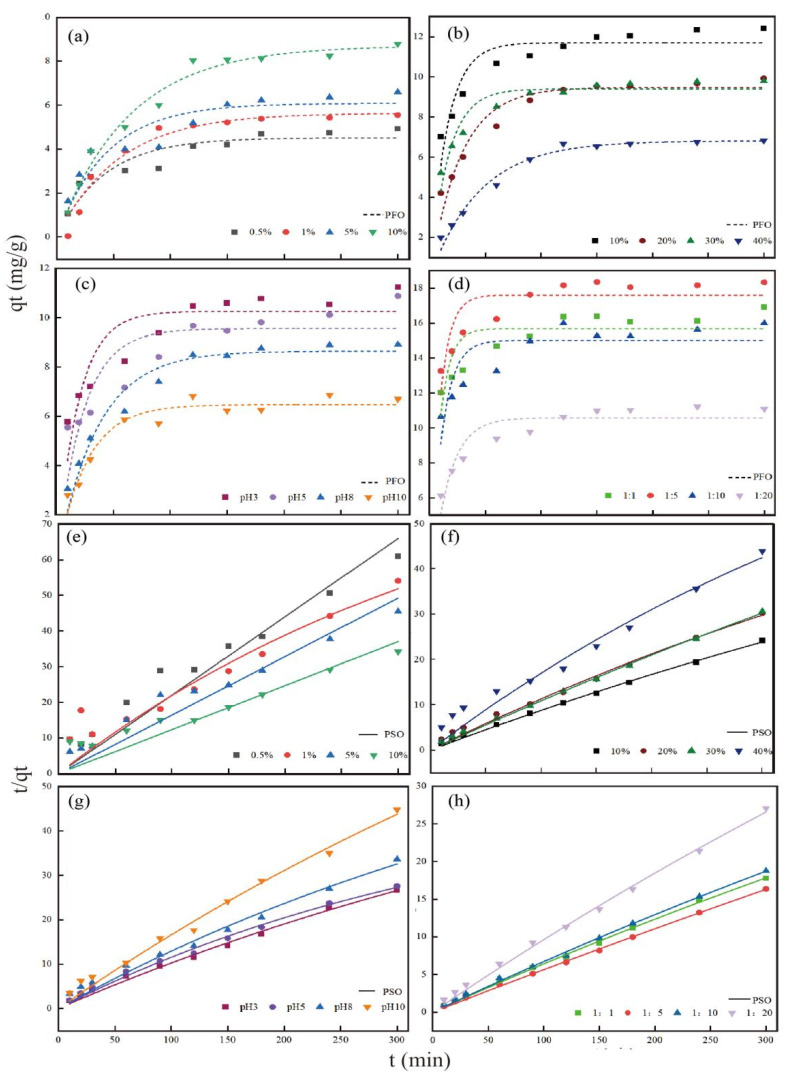
PFO and PSO adsorption models of KWHC: (**a**,**e**) represent salt KWHC in PFO and PSO models, (**b**,**f**) represent oil KWHC in PFO and PSO models, (**c**,**g**) represent pH KWHC in PFO and PSO models, and (**d**,**h**) represent solid-liquid-ratio KWHC in PFO and PSO models.

The fitting results of the adsorption equilibrium time for Cd (II) and the adsorption capacity of KWHC show that both the PFO and PSO models can well fit the adsorption performance of KWHC for Cd (II). The adsorption capacity of KWHC for Cd (II) increases rapidly with time, tends to stabilize after 50 min, and then reaches the adsorption equilibrium (Figure 6). From the analysis of the fitting coefficients of the PFO and PSO models, the PSO fitting coefficient of KWHC is higher than the PFO kinetic fitting coefficient, which indicates that the adsorption process of KWHC for Cd (II) is mainly chemical adsorption (Table 5) [55]. Chemical adsorption results from the chemical bond force between the solid surface of the adsorbent and the adsorbed substance, which greatly exceeds the Van der Waals force of physical adsorption, and the chemical adsorption process is irreversible [56]. Therefore, chemical adsorption is more stable for the fixation of adsorbates than physical adsorption [57]. The adsorption mechanism of KWHC on Cd (II) is chemical adsorption, and its adsorption performance is more stable.

### 4.5. Correlation between Different Parameters and Application Performance

**Figure 7:**
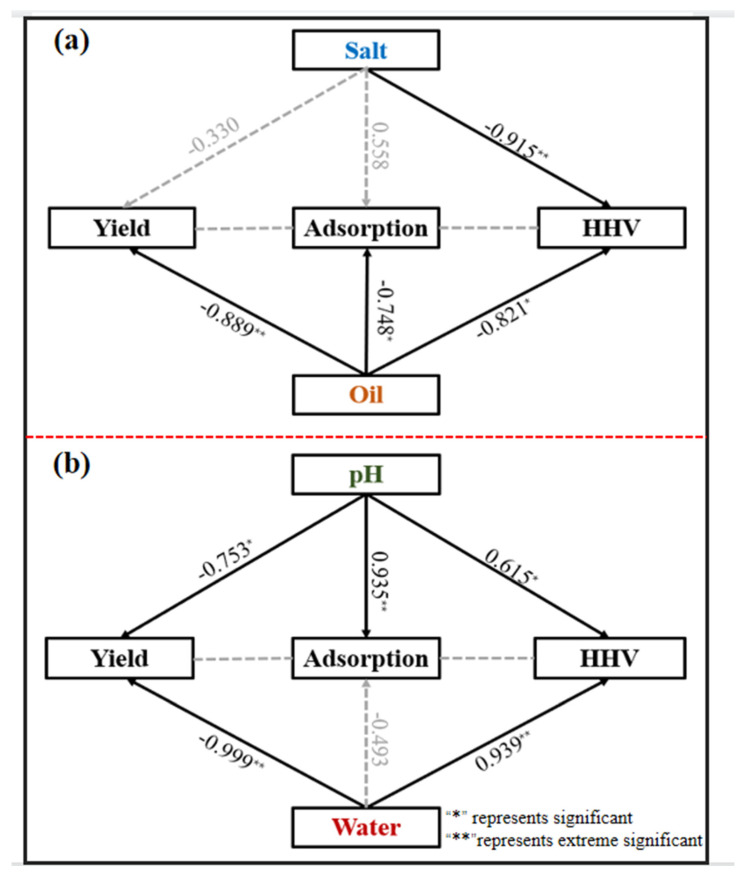
Correlation between treatment parameters and application performance: (**a**) the correlation between salt oil content and KWHC production, Cd (II) adsorption, and HHV value; and (**b**) the correlation between pH and solid liquid ratio and KWHC production, Cd (II) adsorption, and HHV value.

The correlation between the application performance of KWHC and the oil, salt, pH, and solid-liquid ratio was determined by Pearson correlational analysis. The salt did not significantly affect the sorption of KWHC, but it decreased the HHV values. However, the S value of KWHC with high salt content is still more than 2 × 10^−7^, which still has good combustion applicability. Oil negatively impacts the HHV and adsorptive properties of KWHC, but according to actual experimental data, it has a limited inhibitory effect on application performance. The adsorptive capacity and S value are almost at the same level. The HTC can control the negative impact of oil content. The correlation analysis indicated that the pH of the water in the HTC process was positively correlated with adsorption and HHV. Increased water content has no direct effect on the adsorptivity of KWHC and increases the HHV (*p* > 0.05) (Figure 7).

This explored the negative effects of oil salts in KW that HTC can reliably treat, while the application performance of the hydrothermal derivative of KW also does not show obvious limitations on the hydrothermal parameters (pH and moisture). The inclusiveness of the HTC process for raw materials and production process parameters is described.

## 5. Conclusions

This study explored whether the high salt content does not inhibit the Cd (II) adsorption performance of KWHC. The oil content slightly inhibits the Cd (II) adsorption and calorific value of KWHC. The water content has a reduced hydrochar yield compared to the oil, salt, and pH. In addition, the combustion characteristics of KWHC have more stable combustible properties under alkaline conditions. All KWHC treatments were more suitable for combustion after HTC treatment (S values over 2 × 10^−7^). Based on experimental results, it could be speculated that the KWHC can maintain the stability of application performance when the self-reaction mechanism changes the pH. Furthermore, HTC is highly inclusive of raw materials and process parameters because it removed the step of screening sources and special treatment, which expands the feasibility of large-scale industrial production of hydrochar from KW in the future. Based on the findings from this study, it is concluded that the HTC is more suitable and efficient for treating and utilizing KW, thereby achieving a safer and more environmentally friendly disposal method during a national crisis.

## Figures and Tables

**Table 1:** Treatments of the hydrothermal carbonization experiment.

Samples	Rice (g)	Egg (g)	Vegetables (g)	Salt (g)	Oil (g)	Water (g)	pH
S1	9.99 ± 0.02	2.67 ± 0.02	0.67 ± 0.00	0.067 ± 0.00	0.00	66.67 ± 0.01	7
S2	9.99 ± 0.03	2.67 ± 0.00	0.67 ± 0.00	0.13 ± 0.00	0.00	66.67 ± 0.16	7
S3	9.99 ± 0.01	2.67 ± 0.00	0.67 ± 0.00	0.67 ± 0.00	0.00	66.67 ± 0.03	7
S4	9.99 ± 0.01	2.67 ± 0.00	0.67 ± 0.01	1.33 ± 0.00	0.00	66.67 ± 0.03	7
O1	9.99 ± 0.00	2.67 ± 0.02	0.67 ± 0.03	0.00	1.33 ± 0.02	66.67 ± 0.02	7
O2	9.99 ± 0.01	2.67 ± 0.00	0.67 ± 0.00	0.00	2.67 ± 0.02	66.67 ± 0.03	7
O3	9.99 ± 0.00	2.67 ± 0.00	0.67 ± 0.00	0.00	3.99 ± 0.01	66.67 ± 0.01	7
O4	9.99 ± 0.01	2.67 ± 0.00	0.67 ± 0.00	0.00	5.33 ± 0.01	66.67 ± 0.01	7
P1	9.99 ± 0.02	2.67 ± 0.02	0.67 ± 0.01	0.00	0.00	66.67 ± 0.05	3
P2	9.99 ± 0.00	2.67 ± 0.00	0.67 ± 0.01	0.00	0.00	66.67 ± 0.03	5
P3	9.99 ± 0.00	2.67 ± 0.00	0.67 ± 0.01	0.00	0.00	66.67 ± 0.05	8
P4	9.99 ± 0.00	2.67 ± 0.00	0.67 ± 0.01	0.00	0.00	66.67 ± 0.01	10
R1	30.00 ± 0.02	8.00 ± 0.02	2.00 ± 0.02	0.00	0.00	40.00 ± 0.01	7
R2(CK)	9.99 ± 0.02	2.67 ± 0.02	0.67 ± 0.01	0.00	0.00	66.67 ± 0.01	7
R3	5.45 ± 0.01	1.15 ± 0.02	0.36 ± 0.02	0.00	0.00	72.72 ± 0.02	7
R4	2.86 ± 0.01	0.76 ± 0.02	0.19 ± 0.02	0.00	0.00	76.19 ± 0.03	7

**Table 2:** Composition analysis of hydrochar.

	Ultimate Analysis (% d.b)				Atomic Ratio		
Samples	C	N	H	O	H/C	O/C	C/N
S1	64.01	4.69	5.72	16.19	1.072	0.190	15.596
S2	62.13	4.65	5.84	18.78	1.128	0.227	15.913
S3	63.18	4.32	5.56	19.21	1.056	0.228	17.050
S4	59.65	4.07	5.59	18.84	1.125	0.237	17.097
O1	68.80	4.24	7.07	18.56	1.234	0.202	18.940
O2	66.90	3.82	7.18	18.18	1.287	0.204	20.430
O3	67.28	3.82	6.80	18.82	1.212	0.210	20.547
O4	47.33	2.64	4.58	16.97	1.160	0.269	20.913
P1	64.06	5.08	6.30	21.69	1.181	0.254	14.707
P2	62.01	4.52	6.30	19.11	1.220	0.231	16.016
P3	69.73	4.97	5.56	18.32	0.957	0.197	16.374
P4	66.17	4.55	5.78	18.66	1.048	0.212	16.986
R1	65.26	3.03	5.64	21.14	1.036	0.243	25.145
R2 (CK)	63.67	4.27	5.99	20.60	1.129	0.243	17.401
R3	64.47	4.52	6.17	18.22	1.149	0.212	16.626
R4	66.61	5.19	5.90	20.14	1.063	0.227	14.975

**Table 3:** Combustion parameters of the KWHC.

Sample	T_i_ (°C)	T_p_ (°C)	T_b_ (°C)	S (%^2^/°C^−3^∙min^2^)	HHV(MJ/Kg)
S1	246.74	349.98	481.95	6.86 × 10^−7^	26.886
S2	246.52	353.73	505.43	5.01 × 10^−7^	25.969
S3	253.84	352.02	503.87	5.26 × 10^−7^	25.846
S4	256.70	407.13	541.00	4.87 × 10^−7^	24.768
O1	286.38	379.78	468.32	7.74 × 10^−7^	30.014
O2	281.38	379.78	472.32	7.58 × 10^−7^	29.583
O3	280.10	386.93	474.60	5.12 × 10^−7^	29.060
O4	243.92	382.33	543.91	4.93 × 10^−7^	19.488
P1	248.91	357.84	475.68	7.36 × 10^−7^	26.762
P2	247.82	364.92	506.87	5.48 × 10^−7^	26.525
P3	228.98	377.19	504.17	5.85 × 10^−7^	28.217
P4	227.89	377.19	549.33	4.66 × 10^−7^	27.266
R1	278.79	412.44	548.03	4.70 × 10^−7^	26.316
R2(CK)	252.80	349.84	536.76	4.59 × 10^−7^	26.376
R3	273.81	353.44	478.28	6.45 × 10^−7^	27.331
R4	235.26	351.14	500.81	7.10 × 10^−7^	27.330

**Table 4:** Langmuir and Freundlich model parameters.

Treatment	Q_max_ (mg/g)	Langmuir		Freundlich	
		b	R^2^	n	R^2^
S1	4.31	4.06	0.89	0.50	0.80
S2	5.31	7.22	0.92	0.54	0.85
S3	6.02	8.41	0.90	0.52	0.81
S4	8.13	11.14	0.86	0.48	0.76
O1	12.80	11.27	0.98	0.17	0.95
O2	10.93	8.61	0.92	0.15	0.98
O3	10.22	14.98	0.97	0.20	0.94
O4	6.87	7.51	0.93	0.36	0.96
P1	15.16	8.60	0.94	0.15	0.98
P2	7.23	8.19	0.99	0.35	0.88
P3	7.62	9.69	0.88	0.37	0.70
P4	4.10	4.30	0.97	0.31	0.90
R1	16.90	21.22	0.98	0.47	0.90
R2 (CK)	18.33	22.24	1.00	0.43	0.94
R3	15.99	19.86	0.98	0.43	0.90
R4	11.08	12.35	1.00	0.37	0.92

**Table 5:** PFO and PSO model parameters.

Treatment	Q_max_ (mg/g)	Pseudo-First-Order		Pseudo-Second-Order	
		K_1_ (h^−1^)	R^2^	K_2_ (h^−1^)	R^2^
S1	4.92	0.02	0.84	2.28	0.91
S2	5.55	0.02	0.95	0.00	0.87
S3	6.59	0.02	0.85	3.35	0.90
S4	8.78	0.02	0.98	2.90	0.78
O1	12.41	0.06	0.84	0.00	1.00
O2	9.93	0.04	0.93	0.00	0.99
O3	9.80	0.06	0.90	7.96	1.00
O4	6.83	0.02	0.98	0.00	0.95
P1	11.24	0.05	0.77	0.00	0.99
P2	10.88	0.04	0.07	0.00	0.99
P3	8.92	0.03	0.95	0.00	0.98
P4	6.86	0.04	0.93	0.00	0.99
R1	16.90	0.11	0.59	7.85	1.00
R2 (CK)	18.35	0.11	0.68	3.83	1.00
R3	15.99	0.09	0.67	7.65	1.00
R4	11.22	0.06	0.84	8.66	1.00

## Data Availability

Data will be made available on request.
